# How self-compassion fosters resilience in medical sciences students: the protective power of savoring

**DOI:** 10.1186/s12909-026-09802-7

**Published:** 2026-06-25

**Authors:** Majid Sadoughi, Fatemeh Hajian Foroushani

**Affiliations:** 1 Department of Psychology, Faculty of Humanities, University of Kashan, Kashan, Iran; 2 M.A. in Educational Psychology, University of Kashan, Kashan, Iran

**Keywords:** Self-Compassion, Resilience, Savoring, Medical Sciences Students, Psychological Well-Being

## Abstract

**Background:**

Medical sciences students are exposed to intense academic and clinical pressures that can compromise psychological well-being. Promoting resilience in this population is crucial for mitigating the psychological impact of these demands. Self-compassion has been linked to greater resilience, yet the mechanisms underlying this relationship, particularly the role of savoring, remain underexplored. This study examined whether savoring mediates the association between self-compassion and resilience.

**Methods:**

In this cross-sectional correlational study, 310 students from Kashan University of Medical Sciences were selected through stratified random sampling. Participants completed the Connor–Davidson Resilience Scale, the Self-Compassion Scale, and Bryant’s Savoring Beliefs Inventory. Data were analyzed using structural equation modeling in *AMOS-26*.

**Results:**

Self-compassion showed a significant positive association with both savoring and resilience. Savoring was also positively related to resilience. Mediation analyses indicated that savoring partially accounted for the relationship between self-compassion and resilience, supporting its role as an underlying psychological mechanism.

**Conclusions:**

The findings highlight the potential importance of positive emotional processes in understanding resilience among medical sciences students. Interventions that simultaneously strengthen self-compassion and savoring may enhance adaptive functioning in this population.

## Introduction

Medical education, while intellectually demanding, often exposes students to chronic stressors that can undermine their mental health and academic functioning. Global evidence indicates that nearly one-third of medical sciences students experience symptoms of burnout or depression [1], reflecting a pressing concern for their psychological well-being. Given these pressures, identifying protective psychological factors is essential. One of the key factors is resilience, recognized as the ability to cope effectively with adversity and restore psychological equilibrium [2]. Therefore, high resilience is particularly vital for maintaining the mental health of medical sciences students.

Resilience refers to an individual’s capacity to effectively adapt to unexpected conditions and environmental stressors, helping people protect themselves and employ efficient coping strategies when facing difficulties [3]. Rather than a fixed trait, resilience is now widely conceptualized as a dynamic process that can be strengthened through adaptive emotion regulation and supportive psychological resources [4]. Individuals with high levels of resilience can quickly adapt to environmental challenges in occupational, familial, academic, and social domains and return to their baseline functioning and daily life. Compared with others, these individuals possess greater psychological flexibility, demonstrate more effective cognitive and emotional regulation in confronting challenges, and are characteristically more curious [5].

Resilience is particularly important for medical sciences students, who constantly face academic stress, clinical pressures, and premature professional responsibilities [6, 7]. Evidence shows that students in medicine and allied health sciences experience higher levels of burnout, anxiety, and depression compared to students in other disciplines, highlighting the need for preventive psychological interventions such as resilience enhancement [1]. One emerging construct that plays an increasing role in strengthening resilience is self-compassion.

Self-compassion, as one of the core concepts of positive psychology, refers to the ability to approach suffering and negative experiences without harsh self-criticism, enabling individuals to accept their pain and unpleasant experiences as a natural part of life. This construct consists of three components: (1) *self-kindness*, meaning being understanding and gentle with oneself under stress rather than self-critical; (2) *common humanity*, which involves recognizing one’s suffering as part of the shared human experience rather than as an isolated event; and (3) *mindfulness*, defined as adopting an open, receptive, and nonjudgmental stance toward oneself and one’s painful experiences [8]. Self-kindness may foster resilience by reducing self-criticism and facilitating adaptive coping [9, 10], while common humanity can also mitigate feelings of isolation and promote psychological adjustment [11]. Mindfulness, as the regulatory component of self-compassion, supports balanced awareness of experiences [8, 12] and may enhance engagement with positive experiences, thereby facilitating savoring [13]. Together, these components provide a conceptual basis for understanding how self-compassion may contribute to resilience both directly and through positive affective processes. As a human capacity, self-compassion can alleviate distress and promote better coping with life challenges by reducing self-criticism and activating parasympathetic soothing systems [9]. Furthermore, accumulating evidence suggests that self-compassion functions as an adaptive emotion regulation strategy that protects individuals from psychopathology and fosters resilience through greater psychological flexibility and balanced emotional responding [4, 14].

Empirical findings have demonstrated that self-compassion is associated with resilience, such that higher levels of self-compassion are related to greater resilience [15]. Resilience may be influenced by self-compassion for two main reasons. On the one hand, increased self-compassion enhances coping by reducing self-criticism, improving acceptance of difficult circumstances [9, 10], and strengthening social relationships and family support [10]. On the other hand, self-compassion promotes resilience by shaping positive cognitive changes, enhancing inner resources, correcting irrational evaluations, and fostering acceptance of one’s weaknesses and authentic self [16, 17]. In essence, self-compassion contributes to psychological well-being by balancing the emotional system and reducing negative emotions. This, in turn, enhances cognitive and behavioral management and decreases distressing feelings such as depression, anxiety, and stress [18]. Individuals high in self-compassion tend to be more optimistic, less judgmental of themselves, more hopeful, and more flexible [19]. They do not avoid painful emotions, perceive emotional and psychological suffering realistically, and accept it more easily [20]. These individuals can share painful emotions with others. Such acceptance of human experiences facilitates greater life acceptance [11, 21].

Recent studies have shown that self-compassion in medical sciences students can also reduce academic stress and promote resilience by strengthening psychological resources [22, 23]. Despite the recognized importance of self-compassion in enhancing resilience, there is a clear research gap in understanding the mechanisms through which self-compassion influences resilience among Iranian medical sciences students. Most studies have focused only on the direct association between the two constructs, whereas examining mediating variables may provide a foundation for more effective interventions. One important potential mediator is *savoring*—a construct in positive psychology defined as the mental ability to experience, deepen, and sustain positive emotions [13]. Emerging evidence suggests that individuals with higher levels of self-compassion engage more frequently in savoring behaviors [24]. This pattern reflects a cognitive–emotional link between compassionate self-awareness and the capacity to appreciate positive experiences. Research indicates that savoring, especially in stressful situations, can function as an adaptive coping strategy and enhance resilience levels [25, 26].

Since both self-compassion and savoring are conceptually linked to mindfulness, acceptance, emotion regulation, and meaning-making, examining the mediating role of savoring may clarify the psychological mechanisms underlying resilience among medical sciences students. Savoring can relate to positive experiences across temporal domains—past, present, or future. Effective strategies for prolonging positive experiences include sharing with others, reminiscing, self-congratulation, comparison, sensory sharpening, absorption, behavioral expression, temporal awareness, counting blessings, and avoiding “kill-joy thinking” [13]. Numerous studies in positive psychology have provided supporting evidence for the role of savoring in producing diverse positive outcomes through these strategies [13, 27–29].

**Fig. 1:**
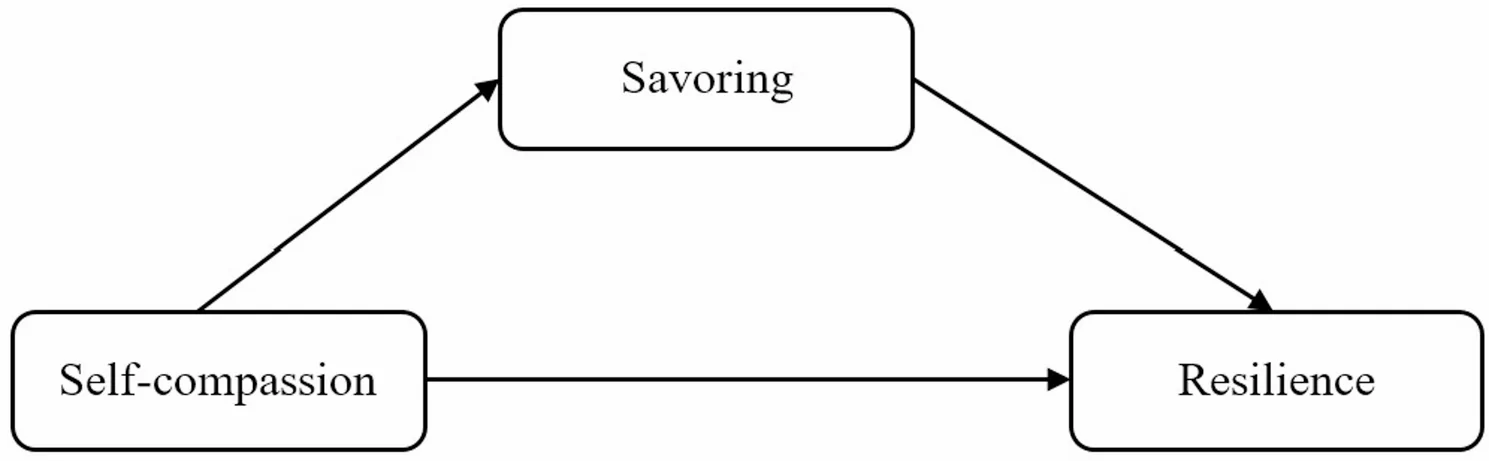
The conceptual model

Given the theoretical importance of understanding the mechanisms linking self-compassion and resilience, the present study examined the mediating role of savoring to clarify how self-compassion influences resilience and to inform interventions aimed at enhancing psychological adaptation. Considering the high levels of psychological pressure, academic stress, and daily exposure to emotionally demanding situations, medical sciences students are particularly vulnerable to emotional exhaustion and compromised mental health [6]. Previous studies have consistently demonstrated a positive association between self-compassion and resilience (e.g. [4, 15]); however, the psychological pathways explaining this link remain insufficiently understood. Rather than re-examining this well-established relationship, the present study extends the literature by exploring savoring—a positive emotion regulation strategy—as a potential mediator. Savoring, or the ability to attend to and prolong positive experiences, may represent a mechanism through which the mindful acceptance inherent in self-compassion translates into active positive emotional engagement. By investigating this mediating process among Iranian medical sciences students—a population frequently exposed to chronic stress and performance pressure—this study provides novel insights into the psychological mechanisms that foster resilience in demanding academic and cultural contexts. Accordingly, this study aimed to test a mediation model (see conceptual model, Fig. 1) in which savoring beliefs mediate the association between self-compassion and resilience among students at Kashan University of Medical Sciences. Specifically, we hypothesized: (H1) Self‑compassion predicts resilience in medical sciences students, and (H2) Savoring mediates the relationship between self‑compassion and resilience.

## Method

### Participants and procedure

This study was applied in purpose and cross-sectional correlational in design. The statistical population included all students of Kashan University of Medical Sciences in 2025. The sample size was determined according to Kline’s recommendations [30]. The proposed research model included 31 free parameters (11 factor loadings, 3 structural paths, and 17 variances). Considering the minimum ratio of 10 observations per parameter, a sample size of 310 participants was determined. The participants (49.4% female; *M*_age_ = 22.80, *SD* = 2.26) were selected using a stratified random sampling method. Specifically, classes from different faculties of Kashan University of Medical Sciences were randomly selected proportionate to the number of students in each faculty and balanced for gender distribution (male/female).

Inclusion criteria included being a student at Kashan University of Medical Sciences and willingness to participate in the study. Exclusion criteria included unwillingness to participate or withdrawal from participation, and incomplete questionnaires (i.e., missing responses to more than 10% of items). Before completing the questionnaires, the purpose of the study was explained to the participants, and they were assured of anonymity and confidentiality of their data. After obtaining informed consent, participants who agreed voluntarily completed the questionnaires. Ethical approval was obtained from the Ethics Committee of Kashan University of Medical Sciences (Approval Code: IR.KAUMS.REC.1404.004). Data were analyzed using structural equation modeling (SEM), and the significance of all direct and indirect effects was evaluated using bias-corrected bootstrap confidence intervals based on 2,000 resamples.

### Instruments

#### Connor–davidson resilience scale – short form

Resilience was assessed using the Connor–Davidson Resilience Scale (CD-RISC) [31]. Although the original short form includes 10 five-point Likert scale items, the Persian validation study [32] supported an 8-item version after removing two poorly performing items. The total score for the 8-item version ranges from 8 to 40, with higher scores indicating greater resilience. The Persian validation study also provided evidence of construct validity based on confirmatory factor analysis, indicating satisfactory model fit, with additional support for convergent validity reflected in positive correlations with self-efficacy and life satisfaction and a negative correlation with aggression [32]. In the Persian validation study, the internal consistency of the 8-item version was α = 0.669 [32]. In the present study, however, the scale demonstrated good internal consistency (Cronbach’s α = 0.859; Table 2).

#### Self-Compassion Scale – Short Form (SCS–SF)

Self-compassion was assessed using the 12-item short version of the Self-Compassion Scale developed by Raes et al. [33]. This instrument is based on Neff’s theoretical model (8) and evaluates three bipolar dimensions: self-kindness vs. self-judgment, mindfulness vs. over-identification, and common humanity vs. Isolation. Respondents rated items on a 5-point Likert scale (1 = almost never, 5 = almost always). Total scores range from 12 to 60, with higher scores reflecting greater self-compassion. Reliability of the Persian version was reported by Shahbazi et al. [34] with Cronbach’s α = 0.91 for the total scale and α ranging from 0.77 to 0.92 across subscales, consistent with the reliability coefficients of the original version (total α = 0.86; subscales 0.55–0.81) [33]. Convergent validity in the Iranian sample was confirmed via significant negative correlations with the General Health Questionnaire (GHQ) (total *r* = − .45, *p* < .001; subscales *r* = − .28 to − 0.48, *p* < .05; [34], which reflects the inverse association between self-compassion and psychological symptoms due to reverse scoring in the GHQ. Construct validity was also confirmed through confirmatory factor analysis (CFI = 0.99, NFI = 0.96, GFI = 0.96, AGFI = 0.91; 28), supporting the six-factor structure. Furthermore, a high correlation (*r* = .97) between the short and long versions of the scale [33] indicates excellent convergent validity.

#### Bryant’s Savoring Beliefs Inventory (SBI)

Savoring beliefs were measured using the 24-item Savoring Beliefs Inventory (SBI) developed by Bryant [35]. The instrument includes three subscales: Anticipation of Future Pleasure, Savoring the Moment, and Reminiscing about Past Pleasure. Responses were rated on a 5-point Likert scale, and total scores range from 24 to 120, with higher scores reflecting stronger savoring beliefs. The Persian version validated by Azimi et al. demonstrated excellent reliability coefficients for subscales: anticipation (α = 0.89), present-moment enjoyment (α = 0.91), and reminiscence (α = 0.84), consistent with the original version (total α = 0.88–0.94, subscales 0.68–0.89; 29, 30). Construct validity was confirmed through confirmatory factor analysis both in the Iranian sample (CFI = 0.945, TLI = 0.938, RMSEA = 0.057, AGFI = 0.861; 30) and in the original study (GFI = 0.82, χ²/df = 3.533; 29). Convergent validity in the original study was established through significant positive correlations with Psychological Well-Being (mean *r* = .493, *p* < .01; 30), Positive Affe**ct** (*r* = .27), Extraversion (*r* = .48), and Optimism (*r* = .42), and negative correlations with Hopelessness (*r* = − .50) and Neuroticism (*r* = − .38) (29). Divergent validity was demonstrated by the nonsignificant correlation with Social Desirability (*r* = − .01), indicating minimal social desirability bias [35].

## Results

### Descriptive statistics and correlations

Of the total participants, 153 (49.4%) were female and 157 (50.6%) were male. The distribution across faculties was as follows: 68 students (21.9%) from the Faculty of Nursing and Midwifery, 39 (12.6%) from the Faculty of Paramedicine, 159 (51.3%) from the Faculty of Medicine, 31 (10%) from the Faculty of Health, and 13 (4.2%) from the Faculty of Dentistry. Participants ranged in age from 18 to 30 years, with a mean age of 22.80 ± 2.26 years.

Table 1 presents the means, standard deviations, skewness, kurtosis, and bivariate correlations among the study variables.

**Table 1:** Correlation Coefficients among the Study Variables

Variables	1	2	3	M ± SD	Skewness	Kurtosis
1) Self-Compassion	-			32.80 ± 7.18	0.019	−0.018
2) Resilience	0.597^**^	-		19.93 ± 4.66	0.200	0.291
3) Savoring	0.493^**^	0.514^**^	-	59.13 ± 14.39	0.116	−0.065

Note: ^**^p < .01

### Measurement model

To evaluate the measurement model, comprehensive assessments of convergent and discriminant validity as well as reliability were performed. Specifically, Confirmatory Factor Analysis (CFA) and Average Variance Extracted (AVE) were used to assess validity, while Cronbach’s alpha and Composite Reliability (CR) were employed to assess reliability.

Table 2 presents the CFA results for first-order factors, estimated using the maximum likelihood method in AMOS, along with the reliability and validity indices for the study variables.

**Table 2:** Results of Confirmatory Factor Analysis, Convergent Validity, and Reliability Indices of Instruments

Variable	χ²	df	χ²/df	*p*	GFI	CFI	RMSEA	AVE	CR	α
Self-Compassion	100.19	47	2.132	< 0.001	0.945	0.953	0.061	0.733	0.892	0.818
Resilience	34.72	20	1.736	0.022	0.974	0.982	0.049	0.504	0.890	0.859
Savoring	445.22	247	1.803	< 0.001	0.893	0.939	0.051	0.778	0.913	0.862
Acceptable	—	—	< 3	< 0.01	> 0.90	> 0.90	< 0.08	> 0.50	> 0.70	> 0.70

*GFI* Goodness-of-Fit Index, *CFI *Comparative Fit Index, *RMSEA *Root Mean Square Error of Approximation, *SRMR *Standardized Root Mean Square Residual, *AVE *Average Variance Extracted, *CR *Composite Reliability

Given the cross-sectional self-report design, common method variance was assessed using Harman’s single-factor test and confirmatory factor analysis. When all items were loaded onto one factor, only 27.32% of the total variance was explained, rejecting the presence of significant CMV. Additionally, a CFA combining all items under one latent factor showed poor fit indices (GFI = 0.552, CFI = 0.612, TLI = 0.593), further confirming that CMV effects were negligible. To estimate effect coefficients and model fit indices, the maximum likelihood estimation method was used. Univariate normality was supported by skewness and kurtosis values within ± 2. Multicollinearity among predictor variables was examined using the Variance Inflation Factor (VIF) and tolerance values. The VIF values ranged from 1.547 to 2.356, confirming the absence of multicollinearity. All assumptions were met.

### Structural model

To examine the hypothesized relationships, the proposed structural model was estimated. Additionally, gender and age were included as observed covariates, with direct paths specified to resilience, to control for their potential confounding effects. The model fit indices were as follows: χ²_(98)_ = 190.63, χ²/*df* = 1.945, GFI = 0.927, CFI = 0.947, TLI = 0.935, RMSEA = 0.055, SRMR = 0.051, and PCLOSE = 0.219. These indices indicate that the proposed model had an acceptable fit with the data. Additionally, the effects of gender and age on resilience were non-significant. Specifically, a χ²/df value below 3, GFI, CFI, and NFI values above 0.90, and RMSEA and SRMR values close to zero all support good model fit.

**Fig. 2:**
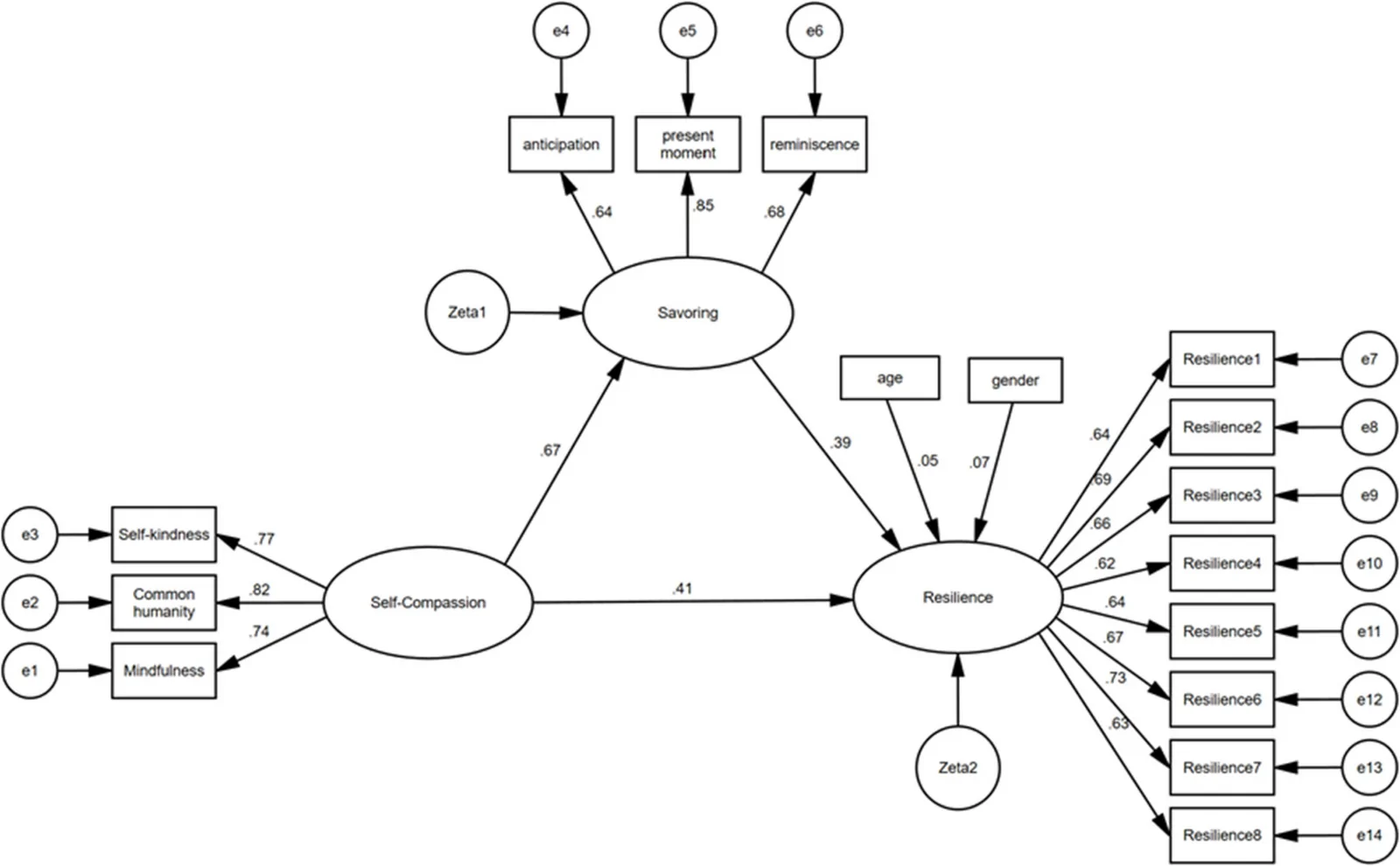
Structural model of the study showing standardized coefficients. Note: All Beta coefficients are significant at .01 level

For the first hypothesis, results indicated that self-compassion significantly predicted both resilience (*β* = 0.415, 95% bias-corrected bootstrap CI [0.223, 0.600], *p* < .001) and savoring (*β* = 0.671, 95% bias-corrected bootstrap CI [0.539, 0.757], *p* < .001). Furthermore, savoring had a positive and significant direct effect on resilience (*β* = 0.395, 95% bias-corrected bootstrap CI [0.229, 0.557], *p* < .001). For the second hypothesis, concerning the mediating role of savoring in the relationship between self-compassion and resilience, results showed that self-compassion indirectly affected resilience through savoring (*β* = 0.265, 95% bias-corrected bootstrap CI [0.143, 0.387], *p* < .001). Furthermore, the total effect of self-compassion on resilience was also significant (*β* = 0.679, 95% bias-corrected bootstrap CI [0.575, 0.771], *p* < .001). Therefore, savoring served as a partial mediator between self-compassion and resilience (see Fig. 2). The model accounted for a substantial proportion of variance in resilience, underscoring the explanatory relevance of the proposed framework. To assess the strength of the mediating effect, the Variance Accounted For (VAF) index was used, representing the ratio of the indirect effect to the total effect. VAF values between 0.20 and 0.80 indicate partial mediation, while values below 0.20 or above 0.80 represent no mediation or full mediation, respectively [36]. In this study, the obtained VAF value was 0.396, confirming partial mediation of savoring in the relationship between self-compassion and resilience among medical sciences students.

## Discussion and conclusion

The present study aimed to examine the mediating role of savoring beliefs in the relationship between self-compassion and resilience among medical sciences students. The findings clearly indicate that self-compassion is associated with resilience both directly and indirectly through the mediating role of savoring. Importantly, the observed partial mediation indicates that savoring represents one significant mechanism—rather than the sole pathway—through which self-compassion contributes to resilience, consistent with process-oriented conceptualizations of resilience as a multidetermined and dynamic construct [2]. The study thereby contributes to the growing body of research on protective psychological processes by integrating two complementary constructs—self-compassion and savoring—within the framework of resilience development. It also provides an empirically supported link between theories of positive emotion regulation and adaptive coping. The level of resilience observed in this sample was somewhat below the theoretical average, which may reflect the high academic and clinical demands commonly reported among medical sciences students. Given that resilience is a modifiable protective factor, this finding underscores the importance of integrating resilience-promoting interventions into medical education curricula.

Consistent with prior research, self-compassion was positively associated with resilience [4, 14, 15, 20, 23]. This consistency across diverse samples strengthens confidence in the robustness of the relationship. Theoretically, self-compassionate individuals are better equipped to maintain psychological equilibrium when facing adversity, partly through superior emotion regulation strategies. Individuals high in self-compassion are less likely to rely on maladaptive strategies such as avoidance or rumination [11]. Instead, they tend to employ adaptive strategies including acceptance of negative emotions, cognitive reappraisal, and mindful awareness. Such adaptive regulation enables reinterpretation of distressing events in less threatening ways, facilitating psychological flexibility and persistence—core components of resilience.

These findings are theoretically aligned with evidence conceptualizing self-compassion as an adaptive emotion regulation mechanism [14, 21]. Trompetter et al. [14], for example, describe self-compassion as a resilience mechanism that buffers against psychopathology through flexible emotional responding. Similarly, Inwood and Ferrari [21] emphasize that self-compassion facilitates adaptive change through balanced emotional processing rather than suppression or overidentification. Within Gilbert’s compassion-focused framework [9], compassion activates affiliative and soothing systems that counteract threat-based emotional responses, which may be particularly relevant in high-performance contexts such as medical education [6, 7].

By viewing negative experiences as temporary and manageable rather than global or uncontrollable, self-compassionate individuals mitigate distress and maintain motivation [37, 38]. For instance, students who respond to academic failures with kindness rather than self-blame are more likely to use setbacks constructively, improving their learning strategies [39]. Thus, self-compassion may serve as a cognitive–emotional buffer that transforms self-criticism into growth-oriented coping, thereby fostering resilience.

Moreover, evidence shows that self-compassion is linked not only to recovery from adversity but also to post-traumatic growth [40], suggesting its transformative potential. From the lens of self-determination theory (SDT), which emphasizes the satisfaction of autonomy, competence, and relatedness as essential for optimal functioning [41], self-compassion may facilitate the fulfillment of these basic needs, particularly relatedness, thereby promoting resilience and growth [42]. Consistent with this, self-compassion has been shown to buffer psychological harm in high-pressure contexts [43]. Taken together, these findings suggest that self-compassion promotes resilience through multiple psychological pathways—emotion regulation, basic need fulfillment, and positive cognitive reappraisal—rather than a single mechanism.

Another key finding of this study was that savoring mediated the relationship between self-compassion and resilience, revealing that the capacity to appreciate and amplify positive experiences functions as an emotional bridge between self-kindness and adaptive coping. The mediating role of savoring highlights a specific emotional-regulatory pathway through which compassionate self-relating may translate into sustained adaptive capacity. Specifically, savoring involves the capacity to attend to, intensify, and prolong positive emotional experiences across temporal domains [13, 35]. Empirical evidence demonstrates that savoring is positively associated with psychological well-being [27, 29] and resilience [26]. Furthermore, recent findings indicate that self-compassion is positively related to savoring tendencies [24], suggesting that individuals who adopt a mindful and nonjudgmental stance toward themselves [8, 44] may be more likely to engage in positive emotional amplification processes [45, 46]. The present mediation results are consistent with this integrative perspective.

Both self-compassion and savoring involve elements of mindfulness [12, 24, 35]. Mindfulness enables individuals to remain aware of difficulties without overidentification or avoidance while simultaneously recognizing positive moments. In this sense, mindfulness operates as the shared attentional mechanism linking self-compassionate awareness of suffering with savoring of joy [8, 44].

Mindfulness is also fundamental to savoring itself, as savoring requires conscious attention to and appreciation of positive experiences [45, 46]. Empirical work indicates that self-compassionate individuals more frequently use savoring strategies, allowing them to sustain positive affect even in stressful circumstances [13, 29]. Such regulation is particularly valuable for medical sciences students who face chronic academic strain. Thus, savoring may amplify the adaptive benefits of self-compassion by broadening attention toward positive experiences and replenishing emotional resources.

From a broader theoretical perspective, the mediating role of savoring is consistent with Fredrickson’s Broaden-and-Build Theory [47]. This theory posits that positive emotions expand cognitive and behavioral repertoires and build enduring resources. Savoring can be understood as a regulatory process that sustains positive affect and contributes to the accumulation of psychological resources relevant to resilience [13, 48]. Prior research supports this: savoring interventions such as focusing on positive moments [49] or amplifying positive emotions [50] have been shown to mitigate stress and promote mental health. Therefore, savoring should be understood not merely as an outcome of positivity but as an active regulatory process that strengthens resilience by cultivating positive affect and psychological flexibility [26, 51, 52].

In summary, the present study provides empirical support for a model in which self-compassion enhances resilience directly and indirectly through savoring. By integrating SDT and the Broaden-and-Build framework, this model illustrates how fulfilling psychological needs and cultivating positive emotions jointly sustain adaptive coping among medical sciences students. Theoretically, the findings advance understanding of resilience as a multifaceted process driven by both self-compassionate self-regulation and positive affective expansion.

### Limitations and recommendations for further studies

While this study offers valuable insights, several limitations should be noted. First, the sample comprised medical sciences students from a single university, which may limit the generalizability of the findings to other cultural and educational contexts. Second, the reliance on self-report data introduces potential bias due to social desirability or self-perception distortions. This may have inflated the observed associations among the study variables, particularly given their shared positive psychological content. Third, the cross-sectional design precludes causal inferences. Accordingly, the directionality of the relationships cannot be established, and reciprocal influences among self-compassion, savoring, and resilience remain possible.

To address these limitations, future research should prioritize longitudinal and experimental designs to clarify temporal ordering and test causal pathways among self-compassion, savoring, and resilience. In addition, cross-cultural replications are needed to evaluate the generalizability of the proposed model across diverse populations and educational contexts. Experimental and intervention-based studies focusing on the enhancement of self-compassion and savoring skills in student and clinical populations would provide stronger causal evidence. Furthermore, incorporating psychophysiological indicators (e.g., heart-rate variability, cortisol) and behavioral measures may help reduce reliance on self-report data and offer a more comprehensive understanding of the underlying affective and regulatory processes.

In practical terms, the findings carry important implications for medical education and student well-being. Given that medical sciences students experience chronic academic and clinical stress, integrating self-compassion and savoring exercises into psychological training programs could enhance resilience and reduce burnout. Structured interventions combining mindful self-kindness (e.g., compassionate self-reflection) with savoring practices (e.g., gratitude journaling, positive reminiscence) could provide cost-effective, evidence-based strategies to enhance psychological well-being and professional sustainability among future healthcare providers. In conclusion, the study underscores that cultivating compassion toward oneself and the capacity to savor positive experiences are mutually reinforcing processes that build resilience. Together, they provide a powerful framework for enhancing emotional endurance in medical education and beyond.

## Conclusion

This study demonstrates that self-compassion contributes to resilience among medical sciences students, both directly and through savoring as a mediating mechanism. These findings underscore savoring as an important pathway linking self-compassion to resilience. From a practical perspective, medical education programs could benefit from incorporating self-compassion training (e.g., compassionate self-reflection, exercises targeting self-kindness) alongside savoring practices (e.g., gratitude journaling, reflection on positive clinical experiences). Embedding such interventions in student counseling, professional workshops, or resilience-focused curricula may reduce burnout, enhance adaptive capacity, and promote long-term well-being among students in high-pressure environments.

## Data Availability

The datasets used and analyzed during the current study are available from the corresponding author upon reasonable request.

## Abbreviations

CFA: Confirmatory Factor Analysis
CFI: Comparative Fit Index
CI: Confidence Interval
CMV: Common Method Variance
GFI: Goodness-of-Fit Index
RMSEA: Root Mean Square Error of Approximation
SEM: Structural Equation Modeling
SRMR: Standardized Root Mean Square Residual
TLI: Tucker-Lewis Index
VAF: Variance Accounted For
α Cronbach’s: Alpha (Internal Consistency Reliability)

## References

[1] Rotenstein LS, Ramos MA, Torre M, Segal JB, Peluso MJ, Guille C, et al. Prevalence of depression, depressive symptoms, and suicidal ideation among medical students: a systematic review and meta-analysis. JAMA. 2016;316(21):2214–36. 27923088 10.1001/jama.2016.17324 PMC5613659

[2] Luthar SS, Cicchetti D, Becker B. The construct of resilience: A critical evaluation and guidelines for future work. Child Dev. 2000;71(3):543–62. 10953923 10.1111/1467-8624.00164 PMC1885202

[3] Maluccio AN, Resilience. A many-splendored construct? Am J Orthopsychiatry. 2002;72(4):596–9.

[4] Hu Y. Examining the effects of teacher self-compassion, emotion regulation, and emotional labor strategies as predictors of teacher resilience in EFL context. Front Psychol. 2023;14:1190837. 37546437 10.3389/fpsyg.2023.1190837 PMC10401064

[5] Block J, Kremen AM. IQ and ego-resiliency: conceptual and empirical connections and separateness. J Personal Soc Psychol. 1996;70(2):349. 10.1037//0022-3514.70.2.349 8636887

[6] Dyrbye LN, Thomas MR, Shanafelt TD. Systematic Review of Depression, Anxiety, and Other Indicators of Psychological Distress Among U.S. and Canadian Medical Students. Acad Med. 2006;81(4):354–73. 16565188 10.1097/00001888-200604000-00009

[7] Tempski P, Santos IS, Mayer FB, Enns SC, Perotta B, Paro HB, et al. Relationship among medical student resilience, educational environment and quality of life. PLoS ONE. 2015;10(6):e0131535. 26121357 10.1371/journal.pone.0131535 PMC4486187

[8] Neff K. Self-compassion: An alternative conceptualization of a healthy attitude toward oneself. Self identity. 2003;2(2):85–101.

[9] Gilbert P. Compassion focused therapy: A special section. Int J Cogn Therapy. 2010.

[10] Alizadeh S, Khanahmadi S, Vedadhir A, Barjasteh S. The relationship between resilience with self-compassion, social support and sense of belonging in women with breast cancer. Asian Pac J cancer prevention: APJCP. 2018;19(9):2469. 10.22034/APJCP.2018.19.9.2469 PMC6249445 30255701

[11] Barnard LK, Curry JF. Self-compassion: Conceptualizations, correlates, & interventions. Rev Gen Psychol. 2011;15(4):289–303.

[12] Brown KW, Ryan RM. The benefits of being present: mindfulness and its role in psychological well-being. J Personal Soc Psychol. 2003;84(4):822. 10.1037/0022-3514.84.4.822 12703651

[13] Bryant FB, Veroff J, Savoring. A new model of positive experience: Psychology; 2017.

[14] Trompetter HR, De Kleine E, Bohlmeijer ET. Why does positive mental health buffer against psychopathology? An exploratory study on self-compassion as a resilience mechanism and adaptive emotion regulation strategy. Cogn therapy Res. 2017;41(3):459–68. 10.1007/s10608-016-9774-0 PMC5410199 28515539

[15] Neff KD, McGehee P. Self-compassion and psychological resilience among adolescents and young adults. Self identity. 2010;9(3):225–40.

[16] Zessin U, Dickhäuser O, Garbade S. The relationship between self-compassion and well‐being: A meta‐analysis. Appl Psychology: Health Well‐Being. 2015;7(3):340–64. 26311196 10.1111/aphw.12051

[17] Neff KD, Germer CK. A pilot study and randomized controlled trial of the mindful self-compassion program. J Clin Psychol. 2013;69(1):28–44. 23070875 10.1002/jclp.21923

[18] Kuchar AL, Neff KD, Mosewich AD. Resilience and Enhancement in Sport, Exercise, & Training (RESET): A brief self-compassion intervention with NCAA student-athletes. Psychol Sport Exerc. 2023;67:102426. 37665879 10.1016/j.psychsport.2023.102426

[19] Yang Y, Zhang M, Kou Y. Self-compassion and life satisfaction: The mediating role of hope. Pers Indiv Differ. 2016;98:91–5.

[20] Javadi A, Manzari Tavakoli A, Manzari Tavakoli H, Zeinaddini Meimand Z. Modeling academic resilience based on self-compassion and positive self-talk concerning the mediating role of academic well-being among secondary school students. J Psychol Sci. 2024;23(141):233–50.

[21] Inwood E, Ferrari M. Mechanisms of change in the relationship between self-compassion, emotion regulation, and mental health: A systematic review. Appl Psychology: Health Well‐Being. 2018;10(2):215–35. 10.1111/aphw.12127 29673093

[22] Beaumont E, Durkin M, Martin CJH, Carson J. Compassion for others, self-compassion, quality of life and mental well-being measures and their association with compassion fatigue and burnout in student midwives: A quantitative survey. Midwifery. 2016;34:239–44. 26628352 10.1016/j.midw.2015.11.002

[23] McArthur M, Mansfield C, Matthew S, Zaki S, Brand C, Andrews J, Hazel S. Resilience in veterinary students and the predictive role of mindfulness and self-compassion. J Vet Med Educ. 2017;44(1):106–15. 28206835 10.3138/jvme.0116-027R1

[24] Schellenberg BJ, Semenchuk B, Bailis DS, Strachan S, Quach A. Good times, bad times: A closer look at the relationship between savoring and self-compassion. Pers Indiv Differ. 2022;198:111807.

[25] Smith JL, Hanni AA. Effects of a savoring intervention on resilience and well-being of older adults. J Appl Gerontol. 2019;38(1):137–52. 28380722 10.1177/0733464817693375

[26] Cheng YC. A hypothetic model for examining the relationship between savoring, imagination, perceived hope and resilience: A study of Taiwanese principals. Psychol Sch. 2024;61(4):1491–513.

[27] Jose PE, Lim BT, Bryant FB. Does savoring increase happiness? A daily diary study. J Posit Psychol. 2012;7(3):176–87.

[28] Hurley DB, Kwon P. Savoring helps most when you have little: Interaction between savoring the moment and uplifts on positive affect and satisfaction with life. J Happiness Stud. 2013;14(4):1261–71.

[29] Smith JL, Bryant FB. Savoring and well-being: Mapping the cognitive-emotional terrain of the happy mind. The happy mind: Cognitive contributions to well-being. Springer; 2017. pp. 139–56.

[30] Kline RB. Principles and practice of structural equation modeling. Guilford; 2023.

[31] Campbell-Sills L, Stein MB. Psychometric analysis and refinement of the connor–davidson resilience scale (CD‐RISC): Validation of a 10‐item measure of resilience. J Trauma Stress: Official Publication Int Soc Trauma Stress Stud. 2007;20(6):1019–28. 10.1002/jts.20271 18157881

[32] Keyhani M, Taghvaei D, Rajabi A, Amirpour B. Internal consistency and confirmatory factor analysis of the Connor-Davidson Resilience Scale (CD-RISC) among nursing female. Iran J Med Educ. 2015;14(10):857–65.

[33] Raes F, Pommier E, Neff KD, Van Gucht D. Construction and factorial validation of a short form of the self-compassion scale. Clin Psychol Psychother. 2011;18(3):250–5. 21584907 10.1002/cpp.702

[34] Shahbazi M, Rajabi G, Maghami E, Jelodari A. Confirmatory factor analysis of the Persian version of the self-compassion rating scale-revised. 2015.

[35] Bryant F. Savoring Beliefs Inventory (SBI): A scale for measuring beliefs about savouring. J mental health. 2003;12(2):175–96.

[36] Sarstedt M, Ringle CM, Hair JF. Partial least squares structural equation modeling. Handbook of market research. Springer; 2021. pp. 587–632.

[37] Diedrich A, Grant M, Hofmann SG, Hiller W, Berking M. Self-compassion as an emotion regulation strategy in major depressive disorder. Behav Res Ther. 2014;58:43–51. 24929927 10.1016/j.brat.2014.05.006

[38] Bluth K, Mullarkey M, Lathren C. Self-compassion: A potential path to adolescent resilience and positive exploration. J Child Fam stud. 2018;27(9):3037–47. 34079199 10.1007/s10826-018-1125-1 PMC8168452

[39] Breines JG, Chen S. Self-compassion increases self-improvement motivation. Pers Soc Psychol Bull. 2012;38(9):1133–43. 22645164 10.1177/0146167212445599

[40] Wong CCY, Yeung NC. Self-compassion and posttraumatic growth: Cognitive processes as mediators. Mindfulness. 2017;8(4):1078–87.

[41] Deci EL, Ryan RM. Self-determination theory: A macrotheory of human motivation, development, and health. Can Psychol. 2008;49(3):182.

[42] Gonzalez-Mendez R, Díaz M. Volunteers’ compassion fatigue, compassion satisfaction, and post-traumatic growth during the SARS-CoV-2 lockdown in Spain: Self-compassion and self-determination as predictors. PLoS ONE. 2021;16(9):e0256854. 34469473 10.1371/journal.pone.0256854 PMC8409613

[43] Winders SJ, Murphy O, Looney K, O’Reilly G. Self-compassion, trauma, and posttraumatic stress disorder: A systematic review. Clin Psychol Psychother. 2020;27(3):300–29. 31986553 10.1002/cpp.2429

[44] Neff KD. The self-compassion scale is a valid and theoretically coherent measure of self-compassion. Mindfulness. 2016;7(1):264–74.

[45] Bryant FB, Smith JL. Appreciating life in the midst of adversity: Savoring in relation to mindfulness, reappraisal, and meaning. Psychol Inq. 2015;26(4):315–21.

[46] Ritchie TD, Bryant FB. Positive state mindfulness: A multidimensional model of mindfulness in relation to positive experience. Int J Wellbeing. 2012;2(3).

[47] Fredrickson BL. The role of positive emotions in positive psychology: The broaden-and-build theory of positive emotions. Am Psychol. 2001;56(3):218. 11315248 10.1037//0003-066x.56.3.218 PMC3122271

[48] Fredrickson BL. The role of positive emotions in positive psychology: The broaden and build theory of positive emotions. Am Psychol. 2001;56(3):218–26. 11315248 10.1037//0003-066x.56.3.218 PMC3122271

[49] McMakin DL, Siegle GJ, Shirk SR. Positive Affect Stimulation and Sustainment (PASS) module for depressed mood: A preliminary investigation of treatment-related effects. Cogn therapy Res. 2011;35(3):217–26. 10.1007/s10608-010-9311-5 PMC3226735 22140287

[50] Krejtz I, Nezlek JB, Michnicka A, Holas P, Rusanowska M. Counting one’s blessings can reduce the impact of daily stress. J Happiness Stud. 2016;17(1):25–39.

[51] Pillay D, Nel P, Van Zyl E. Positive affect and resilience: Exploring the role of self-efficacy and self-regulation. A serial mediation model. SA J Industrial Psychol. 2022;48(1):1–12.

[52] Yang Y-H, Chang S-H, Hua Y-H. Mediating Effect of Principals’ Perceived Workplace Hope on the Relationship Between Workplace Savoring Beliefs and Workplace Resilience. SAGE Open. 2024;14(4):21582440241287008.

